# Chemosensory Cues to Conspecific Emotional Stress Activate Amygdala in Humans

**DOI:** 10.1371/journal.pone.0006415

**Published:** 2009-07-29

**Authors:** Lilianne R. Mujica-Parodi, Helmut H. Strey, Blaise Frederick, Robert Savoy, David Cox, Yevgeny Botanov, Denis Tolkunov, Denis Rubin, Jochen Weber

**Affiliations:** 1 Department of Biomedical Engineering, Stony Brook University School of Medicine, Stony Brook, New York, United States of America; 2 Department of Psychiatry, Stony Brook University School of Medicine; Stony Brook, New York, United States of America; 3 McLean Hospital, Consolidated Department of Psychiatry, Harvard University School of Medicine, Belmont, Massachusetts, United States of America; 4 Athinoula A. Martinos Center for Biomedical Imaging, Massachusetts General Hospital, Department of Radiology, Harvard University School of Medicine, Charlestown, Massachusetts, United States of America; 5 Department of Brain and Cognitive Sciences, Massachusetts Institute of Technology; Cambridge, Massachusetts, United States of America; 6 Department of Psychology, Columbia University; New York, New York, United States of America; Victoria University of Wellington, New Zealand

## Abstract

Alarm substances are airborne chemical signals, released by an individual into the environment, which communicate emotional stress between conspecifics. Here we tested whether humans, like other mammals, are able to detect emotional stress in others by chemosensory cues. Sweat samples collected from individuals undergoing an acute emotional stressor, with exercise as a control, were pooled and presented to a separate group of participants (blind to condition) during four experiments. In an fMRI experiment and its replication, we showed that scanned participants showed amygdala activation in response to samples obtained from donors undergoing an emotional, but not physical, stressor. An odor-discrimination experiment suggested the effect was primarily due to emotional, and not odor, differences between the two stimuli. A fourth experiment investigated behavioral effects, demonstrating that stress samples sharpened emotion-perception of ambiguous facial stimuli. Together, our findings suggest human chemosensory signaling of emotional stress, with neurobiological and behavioral effects.

## Introduction

The existence of alarm substances in communicating emotional stress via chemosensory cues is well-established in mammals [1], with animals exposed to odors secreted by acutely stressed conspecifics expressing neurobiological and behavioral changes consistent with increased arousal and threat-assessment [2]–[4]. In recent years, a significant body of research has explored the role of human chemosensory signals for reproductive function, an area that is controversial [5] but which appears to provide some evidence for influence on humans in some of the same contexts in which they exist for non-human mammals [6]–[18]. This conservation across species is biologically suggestive, and predicts that human chemosensory signals for emotional stress may also exist and assume functional importance.

To date, six studies worldwide have published reports on human stress signaling via sweat. Two studies [19], [20] found that individuals were able to identify, solely by smelling sweat collected on axillary pads, whether the sweat donor had been watching a frightening versus benign film. Using a similar collection paradigm with frightening and benign films, one study [21] found that participants, when smelling the stress, but not neutral, sweat showed improved accuracy in completing a word-association task, while another [22] found that stress sweat caused participants to interpret ambiguous expressions as more fearful. Two studies collected sweat from individuals preparing to take a difficult examination with exercise sweat as the control. In one study, females exposed to the stress odor were less likely to judge a face as positive when primed with a positive face [23], while in the other, auditory stimuli provoked an increased startle response [24] when participants breathed sweat collected during the stress condition.

We set out to determine whether breathing the sweat of people who were emotionally stressed produced, in a group of unrelated individuals, neurobiological evidence of emotion-perception. The primary area associated with emotion-processing is the amygdala [25], [26], which has been reliably activated in human neuroimaging studies of emotion [27] as well as animal studies using rat alarm substances [3].

To obtain human sweat stimuli, we first collected axillary samples obtained from 144 individuals participating in a stress condition (first-time tandem skydive) and a control condition (running on a treadmill for the same duration of time at the same time of day). Sweat donors jumped from 4 km (13,000 ft.), with one full minute of free-fall at a vertical speed of 193 km/hr and four minutes under the parachute. Because the tandem-master controlled the descent, the skydiving condition produced a predominantly emotional but not physical stressor for our sweat donors, while the exercise condition produced a predominantly physical but not emotional stressor. Significant increases in both participant cortisol-levels (repeated-measures ANOVA, *pre−post Stress vs. Exercise*: *F* = 39.87, *p* = 0.000, N = 40) and state-anxiety (paired t-test: *t* = 10.02, *p* = 0.000, N = 40), confirmed that the paradigm was successful at inducing emotional stress. The sweat collection and storage protocols were designed to prevent bacterial growth, which gives otherwise odorless sweat its characteristic aversive odor.

Axillary samples, once extracted and pooled for each condition, were then used as stimuli for four experiments. Two fMRI experiments assessed amygdala activation as well as possible gender interactions that could indicate confounds due to reproductive chemosignals, which have been shown to be sex-specific [13]. The amygdala is not only associated with emotion, but also plays a key role in olfactory processing [28]. To confirm that test and control conditions differed only with respect to emotion, and not perceivable odor, we used a double-blind forced-choice discrimination task, as well as Likert scales, to verify that participants were unable to detect intensity, valence, or qualitative differences in odor between the stress and exercise sweat. Finally, we tested the behavioral implications of the amygdala activation, to investigate how stress sweat affects threat-perception using psychometric curves generated by participants' responses to morphed neutral-to-threatening faces.

**Figure 1:**
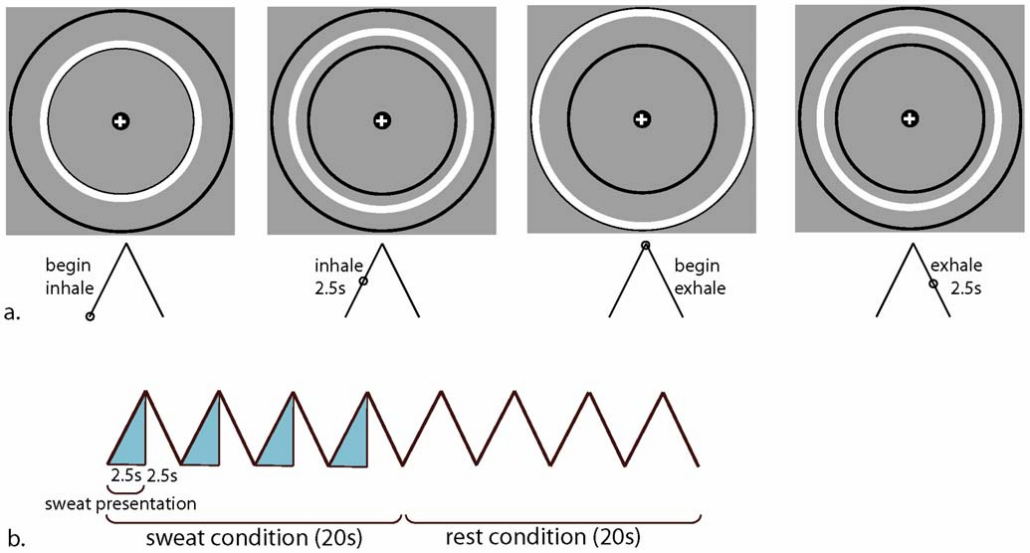
During the fMRI scans, participants' breathing was synchronized via a continuously expanding and contracting circle (a), which cued inhalation and exhalation, respectively. Stress and exercise sweat were presented in a randomized block design, with each 20s block comprised of four inhalations-exhalations (b), timed to a five-second cycle.

Participants for all experiments were screened for anosmia prior to testing. For the fMRI and behavioral experiments, presentation of sweat extracts was controlled with synchronized nasal inhalation (Figure 1); for the odor discrimination experiments, individuals were asked to sniff the sample.

## Results

### fMRI Experiments

**Figure 2:**
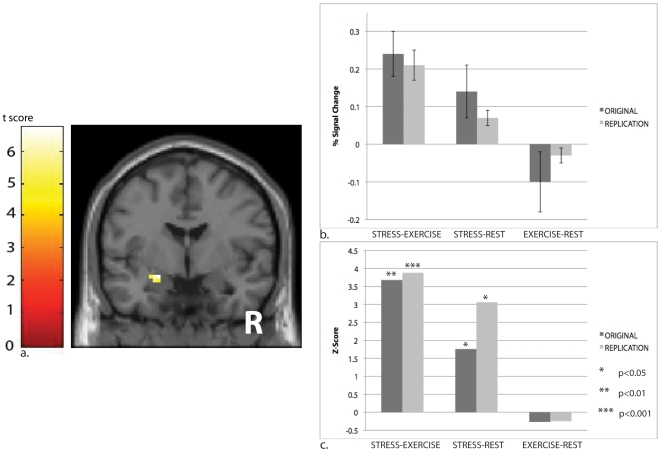
Breathing stress-derived sweat modulates the amygdala, the primary brain region associated with emotional processing. The unmasked activation map *(a)* reflects the STRESS−EXERCISE contrast, and was produced using height threshold *t* = 3.7, *p*<0.001 (uncorrected) and extent threshold k = 5 voxels. The MNI coordinates of the maximally activated voxel, located in the left amygdala, are [*x* = −27, *y* = −6, *z* = −12] (*t* = 5.21/*Z* = 3.88; *p*_(small-volume-corrected)_ = 0.008). Inspection of the mean response to STRESS-REST and EXERCISE-REST contrasts *(b)* initially appeared to suggest mean deactivation in response to EXERCISE sweat. However, once we factored in the variance *(c)*, it became clear that the effect for the STRESS-EXERICISE contrast was predominantly due to activation in response to the STRESS condition, rather than to deactivation in response to the EXERCISE condition, as only the former showed statistically significant changes from baseline.

**Figure 3:**
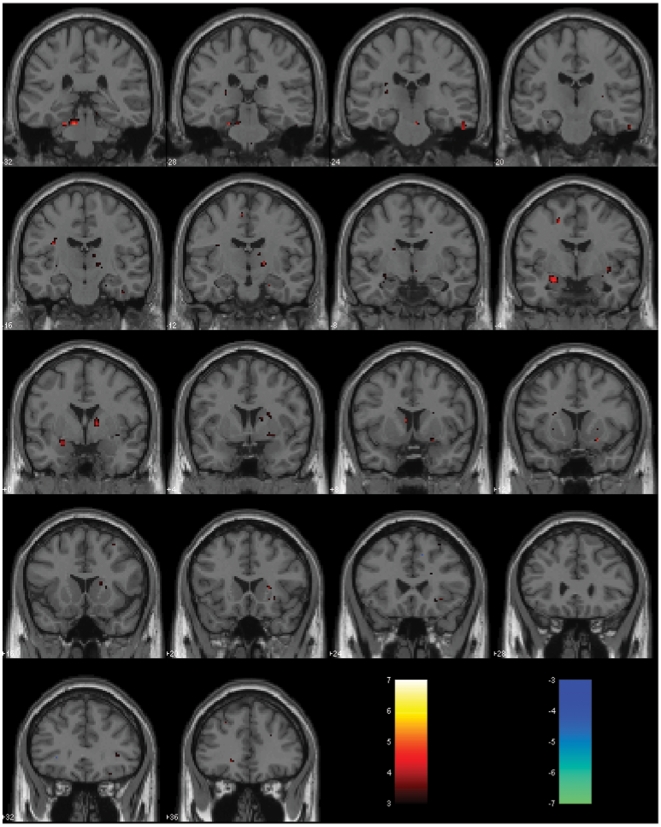
Full-brain activation maps for replication fMRI study, showing activation levels (STRESS>EXERCISE) in warm colors and de-activations (EXERCISE>STRESS) in cool colors, showed that differences between the two conditions were most pronounced in the amygdala, with no significant de-activations. These images were produced at *p*<0.005, with extent threshold = 5 voxels. Table 1 provides a list of all significantly activated clusters corresponding to this whole-brain random-effects analysis.

In the original experiment, we presented sweat from 40 male donors to 16 participants (50% female) while their brains were scanned using fMRI. In a replication experiment, using different participants and scanners, we presented sweat from an additional 40 donors (50% female) to a different group of 16 participants (50% female) undergoing fMRI, increasing power by doubling the number of stimulus presentations. Because we hypothesized that perception of emotional stress would modulate activity in a brain area related to emotion, our analyses focused on the amygdala; all values were corrected for multiple-comparisons using small-volume correction (SVC). For both experiments, these revealed significant activation of the left amygdala (*Original Experiment*: *t* = 4.80/*Z* = 3.68, *p_(svc)_* = 0.02 [MNI *x, y, z* = −16, −10, −18], N = 16; *Replication Experiment*: *t* = 5.21*/Z* = 3.88, *p_(svc)_* = 0.008, [MNI *x, y, z* = −27, −6, −12], N = 16; Figure 2) in response to the stress sweat as compared to the exercise sweat. For both experiments, activity was concentrated most strongly in the superficial, or corticoid, amygdala (*Original Experiment*: *t* = 4.80/*Z* = 3.68, *p_(svc)_* = 0.008, N = 16; *Replication Experiment*: *t* = 5.21*/Z* = 3.88, *p_(svc)_* = 0.008, N = 16)—a region known to have substantial olfactory inputs in primates; homologous structures in other mammals have been implicated in pheromonal processing [29]. Activation patterns were equivalent for same-sex and opposite-sex donor-detector pairs (repeated-measures ANOVA: *Original Experiment*: *F* = 1.76, *p* = 0.21, N = 16; *Replication Experiment*: *Donor Sex*: *F* = 0.21, *p* = 0.65, N = 16; *Detector Sex*: *F* = 1.31, *p* = 0.27, N = 16; Donor Sex*Detector Sex: *F* = 0.004, *p* = 0.952, N = 16), suggesting that reproductive chemosignals, known to be sex-specific in both animals [30] and humans [13], were not the likely cause. Whole-brain random-effects analyses for the STRESS-EXERICISE contrast (Figure 3, Table 1) included the amygdala with no significant de-activations.

**Table 1:** Results of exploratory (random-effects) analysis of STRESS−EXERCISE sweat for replication fMRI study: height threshold T = 3.7 (p_uncorrected_<0.001), extent-threshold = 5 voxels.

MNI Coordinates (x,y,z)	Region	k	T/Z Score	p(uncorr)	Associated Functions
−21,3,−15	Left Amygdala	10	6.19/4.30	0.000	Emotion
−9, −33, −24	Left Cerebellum, Anterior Lobe	7	5.70/4.10	0.000	Integration of sensory perception and motor control
57, −24, −30	Right Inferior Temporal Gyrus (BA20)	6	4.59/3.57	0.000	Visual processing
12,−54,54	Right Precuneus (BA7)	5	4.53/3.54	0.000	Spacial reference system for goal-oriented behavior

There were no significant de-activations.

### Odor Perception Experiments

**Figure 4:**
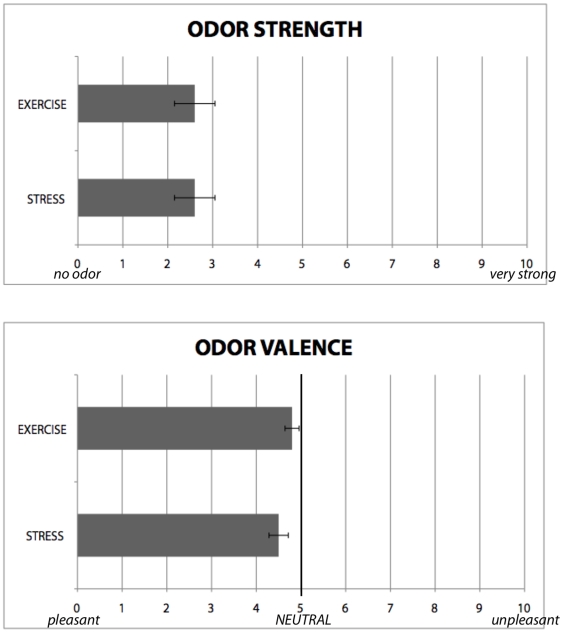
On Likert Scales, participants rated both conditions as mild and neutral; there were no significant differences between their ratings between conditions. A separate forced-choice discrimination experiment additionally indicated that participants were unable to distinguish between the two odors. Together, these suggest that the amygdala activation seen in response to the STRESS, but not EXERCISE, sweat was due to engagement of emotional processing rather than perception of distinct odors.

While the fMRI experiments indicate that participants' amygdala were able to distinguish between the sweat of stressed and non-stressed colleagues, it was important to establish whether this activation might be attributable to odor differences between the two conditions [31], [32]. As shown in Figure 4, subjects rated both odors, using Likert scales ranging from zero (“undetectable”/“pleasant”) to ten (“very strong”/“unpleasant”) as equivalently mild (*Stress*: *μ* = 2.6, s.d. = 2.3, *Exercise*: *μ* = 2.6, s.d. = 2.3; Wilcoxon sign-ranks test: *Z* = 1.11, *p* = 0.28, N = 26) and neutral (*Stress*: *μ* = 4.5, s.d. = 1.1, *Exercise*: *μ* = 4.8, s.d. = 0.8; Wilcoxon sign-ranks test: *Z* = 1.56, *p* = 0.12, N = 26). To investigate whether the conditions had odors that were qualitatively distinct, we also conducted a double-blind forced-choice odor discrimination experiment, in which 16 participants (50% female) identified whether 16 test and control pairs (50% different), randomly presented, were identical or different; participant ratings were not significantly different than chance (one-sample t-test: *t* = 0.64, *p* = 0.53, N = 16). The data suggest that participants were not able to consciously distinguish between test and control odors, and therefore rule out simple odor discrimination as an explanation for amygdala activation in response to the STRESS−EXERCISE contrast.

### Threat-Perception Experiment

**Figure 5:**
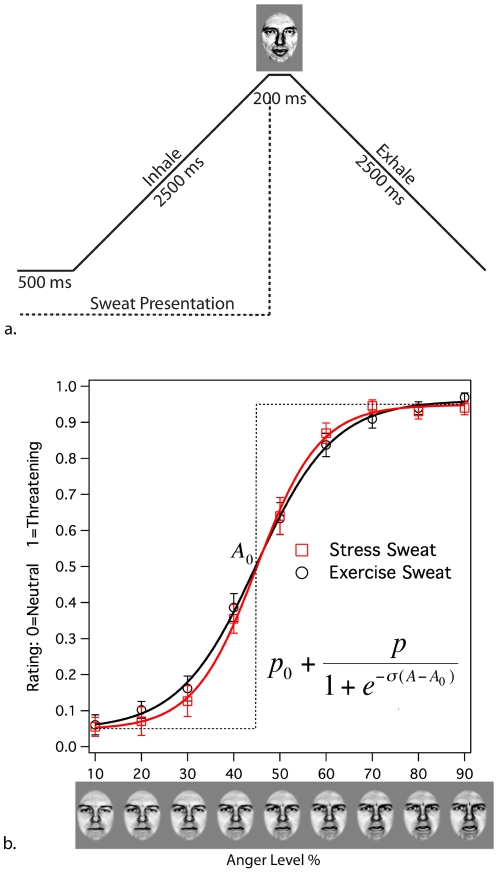
Psychometric curves generated by a forced-choice assessment of ambiguous threat show sharpened discrimination between threat and non-threat while breathing stress-derived sweat. For each participant, data for each condition (STRESS, EXERCISE) were fitted with the sigmoid function, where *p*_0_ and *p*_0_+Δ*p* define upper and lower asymptotes, A_0_ is the inflection point, and σ defines slope. Significant differences between conditions were seen for slope, with individuals under the STRESS condition more closely approximating ideal perceptual discrimination, shown by the dotted line.

Since data from our two previous experiments suggested that the observed amygdala activation reflected emotion discrimination rather than odor discrimination, we then tested whether breathing stress sweat vs. exercise sweat from 64 donors (50% female) behaviorally affected perception of subtle emotional cues in the evaluation of ambiguous faces. Psychometric curves [33] were generated from a forced-choice design in which 14 participants (36% female) indicated via a computer mouse whether briefly-presented (200 ms) male faces, morphed between neutral and angry expressions, were “more neutral” or “more threatening.” For each participant, stress and exercise conditions produced psychometric curves, each composed of nine points ranging from neutral (10%) to angry (90%), with each point the average of 14 face presentations. Threat-levels were presented randomly, with experimental conditions counter-balanced for order. Values for slope, σ, were calculated for each curve using sigmoidal fitting. These showed sharpened discrimination (mean 43% increase) between neutral versus angry faces in response to the stress sweat (*Stress*: *σ = *0.192, s.d. = 0.101; *Exercise*: *σ = *0.134, s.d. = 0.066; repeated-measures ANOVA: *F* = 8.30, *p* = 0.01, N = 14, Figure 5b). No differences between conditions were observed for inflection-points (*F* = 1.35, *p* = 0.27, N = 14), suggesting that the effect was specific to reducing perceptual noise and thereby increasing accuracy in the evaluation of ambiguous threat, rather than to the attribution of threat to neutral stimuli.

## Discussion

While it is commonly known that information regarding the emotional stress of others is communicated in humans by visual and auditory cues, our findings suggest that humans—like other mammals—may complement this information with chemosensory cues as well. Sweat collected during an acute emotional stressor, and subsequently presented to an unrelated group of individuals, produced significant brain activation in regions responsible for emotional processing without conscious perception of distinct odor; behavioral data, our own as well as those from previous studies, suggest the emotional processing may be specific to enhancing vigilance and sharpening threat-discrimination.

Our hypothesis and analyses targeted the amygdala, given its critical role in emotion processing; however, areas associated with vision, motor control, and goal-directed behavior also activated in response to the stress sweat. Previous research has established that emotional stimuli not only activate areas of the brain associated specifically with emotion-perception, but also activate sensory areas associated with perception of concomitantly-presented stimuli [34]; this is thought to reflect the increased salience attached to stimuli perceived within emotional contexts. We therefore suspect that increased activation within the cerebellum, BA7, and BA20 most likely resulted from participants‘ enhanced perception during the stress condition of the visual breathing cues (Figure 1a), which required timing inhalation and exhalation to the motion of expanding and contracting rings throughout the experiment.

Because this was the first neuroimaging study to investigate chemosensory cues to emotional stress, we were careful to rigorously control for a number of potential confounds, both methodological and conceptual. Bacterial contamination of sweat contributes to its strong aversive odor; therefore, we developed sample collection methods that would keep the samples as sterile as possible while still preserving chemical components of interest in apocrine sweat. These were validated using gas chromatography-mass spectroscopy (see Materials and Methods). To ensure that differences observed between the two conditions were not due to differences in participant compliance in following the synchronized cues, we also analyzed trial-specific respiratory parameters for the first experiment (see Materials and Methods) and closely monitored participants’ respiration in real-time during each subsequent experiment. The lack of donor sex-detector sex interactions suggests that the effect is unlikely to be consequent to reproductive pheromones released during either of the two conditions. This is a critical point, since a serious limitation of previous studies using stress sweat was the tendency to use male donors and female detectors, which made it impossible to identify sex-effects or eliminate reproductive pheromones as possible confounds to the effect. Finally, replication of the neurobiological findings across two independent fMRI studies with different donor and detector participants suggests the effect is robust to individual variability.

The mean percentage signal change values (Figure 2b) initially appeared to suggest that, as much as stress sweat increased amygdala activity from baseline, exercise sweat reduced it from baseline; therefore, the effect might have been inflated by our choice of a control condition (although using AIR as a control condition would have been even more problematic since AIR, unlike EXERCISE, would not have controlled for sweat odor). However, statistical analyses that consider the variance (Figure 2c) make clear that it was the STRESS condition, and not the EXERCISE condition, that was primarily responsible for the STRESS-EXERCISE effect. For the original fMRI study, the change for STRESS—REST was statistically significant or trend, whether it was calculated using the maximally-activated voxel (*t* = 1.88/*Z* = 1.76, *p* = 0.04, N = 16), ROI analysis for the superficial amygdala (*t* = 1.48/*Z* = 1.41, *p* = 0.08, N = 16), or ROI analysis for the whole amygdala (*t* = 1.65/*Z* = 1.55, *p* = 0.06, N = 16). However, for the EXERCISE-REST contrast, none of the three was statistically significant (for SVC maximally-activated voxel: *t* = −1.61/*Z* = −0.27, *p* = 0.61, N = 16; for the superficial amygdala ROI: *t* = −0.52/*Z* = 0.52, *p* = 0.30, N = 16; for the left amygdala ROI: *t* = 0.30/*Z* = 0.31, *p* = 0.38, N = 16). Exactly the same pattern held for the replication study. Here, the change for STRESS—REST was even stronger, whether it was calculated it using the maximally-activated voxel (*t* = 3.69/*Z* = 3.06, *p* = 0.001, N = 16), ROI analysis for the superficial amygdala (*t* = 3.23/*Z* = 2.75, *p* = 0.003, N = 16), or ROI analysis for the whole amygdala (*t* = 2.58/*Z* = 2.29, *p* = 0.01, N = 16). However, for the EXERCISE-REST contrast, again none of the three was statistically significant (for SVC maximally-activated voxel: *t* = −1.43/*Z* = −0.25, *p* = 0.59, N = 16; for the superficial amygdala ROI: *t* = 0.36/*Z* = 0.35, *p* = 0.36, N = 16; for the left amygdala ROI: *t* = 0.67/*Z* = 0.66, *p* = 0.26, N = 16). Since both original and replication studies show significant differences for the STRESS-REST contrasts, but not for the EXERCISE-REST contrast, it is clear that results obtained for the STRESS-EXERCISE contrast were not driven by participants' responses to the EXERCISE sweat.

The behavioral effect of the STRESS sweat was to sharpen emotional discrimination, rather than to lower thresholds for attribution of threat. Our findings are in line with more recent conceptualization of the amygdala's role, in which the amygdala appears to be not simply a marker for fear, but rather involved in evaluating stimuli for potential threat and then coordinating appropriate responses via its cortical feedback connections (see, for example, [35]). The latter view is consistent with a wide range of fMRI results: for example, the amygdala is activated during conditioning to pain [36]–[38], anticipation of potential pain ([39] but not to pain itself [40]–[42]; likewise, the amygdala is activated in response to social cues to potential threat, such as the aversive outcomes implied by fearful faces [27] but not to unambiguously threatening stimuli such as the object of phobias [43], [44]. As such, one would expect that a chemosensory cue that facilitates the evaluation and discrimination of threat from non-threat would also activate the amygdala, as well as lowering sensory gating for olfactory, visual, and auditory cues that might further inform risk-assessment.

One potential limitation of our study design was that we morphed between only two facial expressions (fear versus neutral); therefore, our study could not confirm whether the sharpened discrimination that we observed extended to all emotional expressions or was restricted specifically for threat. However, results obtained by a recent study [22] argue against generalization. Asked to distinguish between “happy” and “fearful,” in a design similar to ours using morphed facial expressions, participants rated ambiguous faces as “fearful” more frequently in the context of stress sweat, thereby lowering thresholds for detecting fear in others rather than sharpening discrimination. These results suggest that angry and fearful faces communicate distinct types of information that may interact with chemosensory stress cues in complementary ways. *Angry* faces represent a direct threat, and therefore detection of an anxious colleague's alarm cues may elicit greater vigilance in evaluating whether stimuli signal potential for danger. In contrast, when asked to identify whether faces are *fearful* in the context of stress sweat, participants are essentially integrating multi-modal sensory cues in detecting colleagues' anxiety, much as auditory cues such as laughter would bias visual perception of an ambiguous smiling faces towards “joy.” Future research, using a within-subjects design, can more directly test this hypothesis.

Previous protocols have sampled sweat in response to stressors such as horror films and pre-examination anxiety. These stressors obviously have the advantage of being easier to administer, but are quite removed from alarm pheromones' evolutionary purpose; i.e., fear associated with imminent physical danger. We chose to address this limitation by using first-time tandem skydives, which have shown to reliably induce acute fear (approaching near-pathological states and including traumatic psychological symptoms such as dissociation, loss of awareness, and time-distortion[45]–[53]), in an ethically acceptable and scientifically-controlled manner. The endocrine and self-report measures confirm that the protocol reliably provoked profound emotional stress in our sweat donors. However, debriefing of our donors and their tandem-masters post-jump indicated that while fear markedly increased during the ascent, peaking in the minutes leading up to exiting the plane and during freefall (≥16 minutes), feelings of relief and/or thrill sometimes followed once the parachute opened and upon landing (≤4 minutes). Donor sweat pads could not be removed until immediately after landing; therefore, it is theoretically possible that our neurobiological and behavioral results resulted from chemosignals emitted in response to non-affect-specific hyper-arousal or thrill, rather than pure fear. However, it is important to note that while alarm substances are well-established neurobiologically, behaviorally, and chemically in a wide number of species, including mammals [54], and therefore their conservation in humans is a reasonable extension, an equivalent “thrill” pheromone has never been reported for any species. Therefore, we believe it is much more likely that participants excreted an alarm substance during the initial fear portion of the protocol, which was retained in the sample even in the face of later relief.

## Materials and Methods

All experiments reported in this manuscript were approved by the Institutional Review Board of Stony Brook University. In addition, the replication fMRI experiment was approved by the Institutional Review Board of McLean Hospital; all participants provided written informed consent.

### Methods for Generating Sweat Samples

We recruited 144 participants (“Donors”), each of whom had previously contacted Skydive Long Island (Calverton, NY) to schedule his or her first-time tandem skydive. All participants were between the ages of 18–50 (μ = 25; s.d. = 6), with a Body Mass Index<25, free of cardiac illness, and had not skydived before. Participants wore a digital altimeter (Altimaster Neptune), showing a consistent rise-time of 15 minutes, jump at 13,000 ft, freefall lasting 60 seconds, and parachuting for an additional 4 minutes before landing; this resulted in stress condition of total of 20 minutes (5 minute fall plus 15 minutes of anticipatory anxiety preceding the jump). Salivary cortisol samples were obtained from 40 of the participants using the passive drool method [55] immediately prior to take-off (15 minutes prior to the jump) and again following landing. Cortisol measurements were taken between 1–7 pm to minimize diurnal variability and assayed using Salimetrics Salivary Cortisol Kits (Salimetrics, State College PA). Self-reports of skydiver state anxiety were obtained using the Spielberger State Anxiety Inventory (Mind Garden Inc., Menlo Park, CA), 15 minutes prior to the jump as well as 15 minutes prior to the exercise.

The control condition was conducted on a separate day than the skydive, within 2 hours of the jump-time. During the control condition, each sweat donor was instructed to run on a treadmill at his or her maximum comfortable rate for 20 minutes. We allowed participants to control their own levels of exertion to ensure production of sweat without inducing emotional distress. Salivary samples were obtained immediately prior to start and immediately following the end of the exercise, and were collected and assayed identically to those collected during the stress condition.

For our skydiving condition we deliberately chose to use only tandem jumps. This meant that the tandem-master took over all physical aspects of the jump, including stabilization and steering, to ensure that physiological measures obtained during skydive condition reflected predominantly emotional, and not physical, stress. Our donor participants reported a significant increase in state anxiety (paired t-test: *t* = 10.02, *p* = 0.000, N = 40) between the stress (73^rd^ percentile rank for males 19–39; μ = 42, s.d. = 11) and exercise (25^th^ percentile rank for males 19–29; μ = 28, s.d. = 8) conditions. Mean cortisol values for the stress condition were PRE = 0.229 µG/dL (s.d. = 0.148) and POST = 0.584 µG/dL (s.d. = 0.310), and showed a significant increase (paired t-test: *t* = 7.15, *p* = 0.000, N = 40). Mean cortisol values for the exercise condition were PRE = 0.170 µG/dL (s.d. = 0.165) and POST = 0.207 µG/dL (s.d. = 0.175); no significant increase was observed (paired t-test: *t* = 1.40, *p* = 0.17, N = 40). We additionally performed a repeated-measures ANOVA, comparing PRE v. POST increases between the two conditions. The condition*(PRE, POST) interaction was significant, with the stress condition producing a significantly larger increase (PRE, POST) than the exercise condition (*F* = 39.87, *p* = 0.000, N = 40). Thus, using both self-report and physiological measures we confirmed that skydives were an effective means of inducing a reliable emotional stress response and that exercise functioned as an acceptable control condition.

Sweat sample collection methods were identical for test and control collections. Sweat pads were attached immediately prior to participants' boarding and were removed immediately after landing. The total period of sweat collection, during both skydive and exercise conditions, was 20 minutes. Potential participants were excluded from participation in the study if they had used deodorant/antiperspirant on either day of the sample collection. Participants first had their underarms closely shaved. Prior to application of the sweat pads, the participants' axillary regions were washed with a non-ionic detergent (0.1% Triton X-100), rinsed with ultra-pure water, dried, and finally, washed with isopropanol. Axillary sweat was collected on sterile cleaned (washed 2x in Methanol, 2x in Hexane; both solvents are 99.9% GC^2^, Burdick & Jackson, USA) woven gauze sponges (2×2”, Dukal, USA) that were placed on clean thin mylar squares and taped in the underarm with waterproof adhesive tape (2”, HYTAPE, USA). Solvents were fully evaporated prior to sweat pad construction. After the collection of the sweat was completed, the sweat pad was removed and immediately frozen in tightly sealed Teflon-lined and pre-cleaned borosilicate vials (20 ml, VWR Traceclean, VWR, USA) at −20°C, until the sweat extraction.

To extract the liquid sweat from the sweat pad, we used salivette tubes (Sarstedt, Newton, NC), substituting the sweat pad for the salivette's absorbent insert. Next, double-distilled water was added so that the tube insert was completely filled with both water and the pad. The salivette tubes were placed into an ultrasound bath for 5 minutes and centrifuged to separate the aqueous sample from the cotton pad. The samples were separated into two batches of 20 participants each, each batch of which were pooled and diluted to produce sufficient sample for 8 fMRI experiments. After removal from the sweat pad, the sweat samples were frozen at −20°*C* in 18 ml batches (one per experiment) to avoid multiple thaws. Three hours prior to each fMRI experiment, the samples were thawed at room temperature.

### Validation of Sample Collection Methods

Given that the majority of the compounds detectable in human sweat are hydrophobic [56], [57] but that our method used water to both remove the sweat and act as a medium to present the sweat molecules to the participants, we performed gas chromatography mass spectroscopy as well as calculations using Henry's law to ensure that our collection, extraction, and delivery methods were, in fact, capable of presenting sweat molecules of interest.

#### Analysis of sweat extracts by Gas Chromatography Mass Spectroscopy (GCMS)

GCMS was performed using a VG Quattro triple quadrupole mass spectrometer with EI source and HP5970 gas chromatograph. The samples were taken from males participating in the exercise condition, and were first prepared as for the fMRI experiments (extraction into water). We then added 1∶20th of GC purity hexane (containing 0.3 ng/ml chrysene-d12 for normalization) to partition volatile and semi-volatile molecules into the organic phase. Using a syringe, we recovered 350 µl of hexane that was then blown down with nitrogen to 35 µl of which we injected a few µl into the GCMS. We used a 25m DB5 column for GC. The protocol for GC was as follows: 1) the temperature was kept at 70C for 5 min; 2) we increased the temperature to 300C ramping by 10 C/min; 3) temperature was kept at 300C for ten minutes.

**Figure 6:**
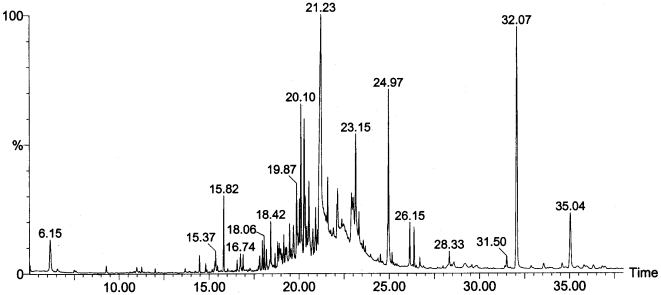
Gas chromatography mass spectroscopy analyses of exercise sweat samples were used to validate that our collection and aqueous extraction methods were capable of sampling over hydrophobic (steroid) components in human apocrine sweat. Total ion count gas chromatography trace of aqueous human sweat extract shows the presence of cholesterol, which is hydrophobic.

**Figure 7:**
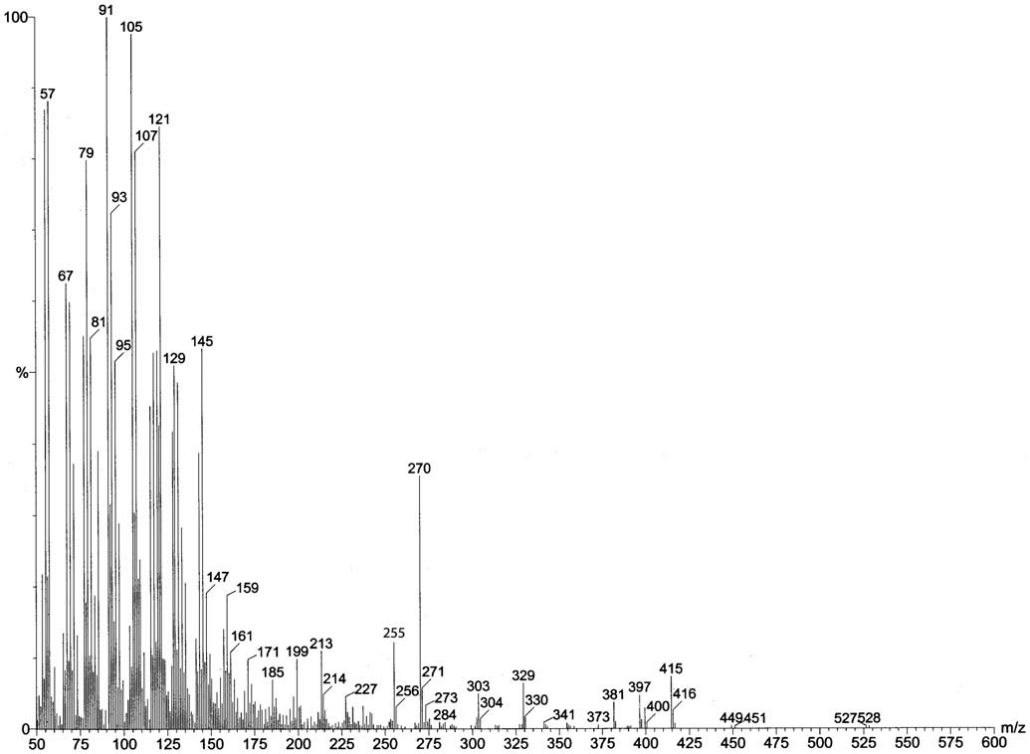
Gas chromatography mass spectroscopy analyses of exercise sweat samples were used to validate that our collection and aqueous extraction methods were capable of sampling over hydrophobic (steroid) components in human apocrine sweat. Mass spectrum of retention time 19.512 minutes shows the presence of human steroids found in apocrine sweat.

**Figure 8:**
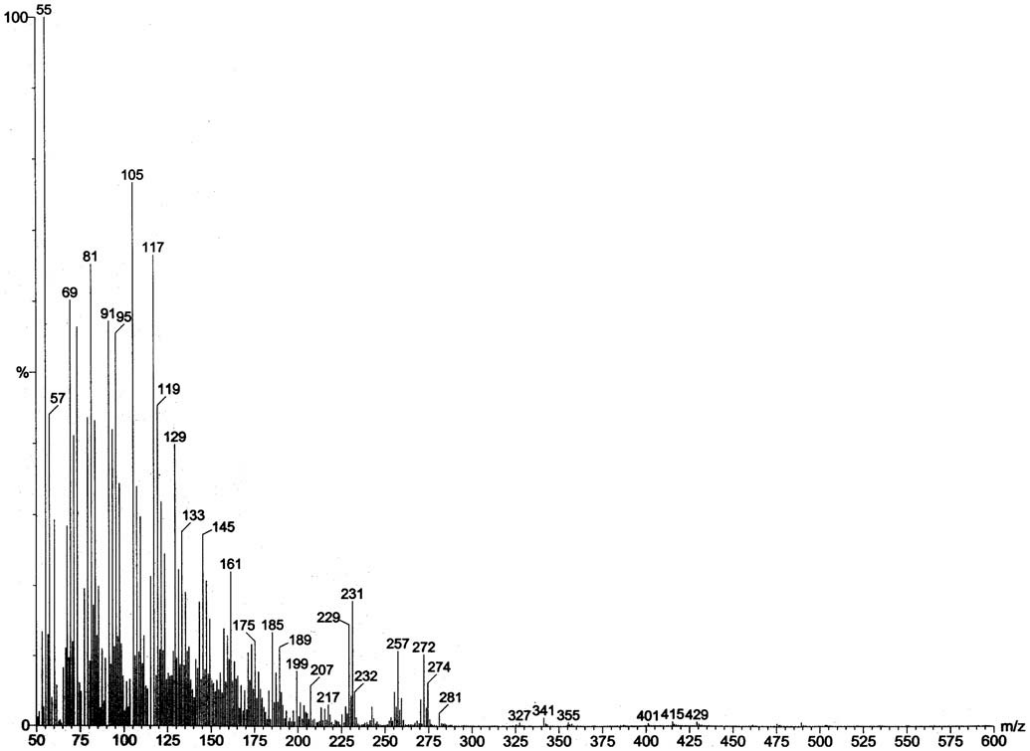
Gas chromatography mass spectroscopy analyses of exercise sweat samples were used to validate that our collection and aqueous extraction methods were capable of sampling over hydrophobic (steroid) components in human apocrine sweat. Mass spectrum of retention time 20.655 minutes shows the presence of human steroids found in apocrine sweat.

A typical total ion count GC trace of our samples is shown in Figure 6. Cholesterol comes off the column at 32.07 min and chrysene-d12 comes off at 26.15 min. The GC scan alone demonstrates that our sweat preparation method yielded quite a few hydrophobic molecules, including cholesterol. In Figures 7–8, we show two individual mass spectroscopy scans that focus on molecular ranges around 270. Since some the odorous steroids (e.g. androst-2-en-17-one MW 272.47, androsta-4,16,dien-3-one MW 270, and androstenone MW 272.42) are in this range we selected GC peaks that show m/z 270 and 272. Comparing the mass spectra below to spectra of pure steroid compounds it is apparent that we clearly have androgen steroids in our samples as judged from the distribution of 270, 255 and 237 fragments and 272, 257 and 229 fragments [58]. The potential candidates for these spectra are androstadienones (MW 272) and androstenones (MW 270), which are of the class of compounds associated with putative human reproductive pheromones, found in apocrine sweat.

#### Release of semi-volatile components from aqueous solutions

Many research olfactometers deliver odors by bubbling air through a liquid containing the fragrance molecules. In our study we paid particular attention to optimizing the delivery of semi-volatile molecules because we expect that a putative stress pheromone will be a steroid derivative (such as androsta-4,16-dien-3-one). The physical chemistry of vaporization of solutes in a typical olfactometer is straightforward. While the bubbles travel through the solution the solutes partition into the air and will be carried away [59]. The degree to which they partition is given by Henry's law:
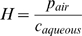
(1)where p_air_ is the partial pressure of the solute in air, c_aqueous_ is the concentration of the solute in water and H is the Henry's law constant. The concentration of a solute with Henry's constant H in a nebulizer will vary in time in the following way [59]:
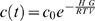
(2)where c_0_ is the initial concentration, G is the air flow rate, and V is volume of the solution (in our case G = 3 l/min and V = 6 ml). Henry's law constants are usually estimated using the boiling point of a substance and its aqueous solubility [60]. For androstenone (C_19_H_28_O) an aqueous solubility of 0.23 mg/l and an air/water partition coefficient of 0.012 is reported [61]. From that we can estimate an exponential decay time for androstenone in our nebulizer setup of about 10 seconds. For more realistic odorous steroids with higher solubility (androst-2-en-17-one c_S_ = 2.3 mg/l, androsta-4,16,dien-3-one c_S_ = 2.9 mg/l [62]) the release properties of our setup (assuming that the partial vapor pressure stays constant) the exponential decay time becomes about 100 seconds. For our nebulizer parameters, and assuming a decay time of 100 seconds, we deliver over the time of the experiment (50 seconds of air flow through the nebulizer) about 40% of the semi-volatile solute.

The GCMS analysis of our sweat preparation and an estimation of Henry's law constants for candidate compounds for a stress pheromone demonstrate that the sweat collection, aqueous extraction, and delivery methods were capable of sampling over hydrophobic pheromone-type compounds in human apocrine sweat. In general, for an efficient delivery one needs to consider all parameters in eq.2. The Henry's law constant for a particular solute can be adjusted by the choice of solvent. To deliver a hydrophobic molecule, the Henry's law constant is highest the more water-like the solvent is. Other parameters that can be adjusted are flow rate and the total volume. If one has fragrance molecules in abundance, the best strategy is to have a long exponential decay time which results in a nearly constant rate delivery. In our case, since both the chemical nature of the molecules and their concentration were unknown variables, we chose the most efficient delivery though an aqueous solution.

### Methods for Presenting Sweat Samples

For all experiments described here, the STRESS (test) and EXERCISE (control) conditions were obtained from pooled sweat obtained from the Donor stress and exercise conditions (described above), respectively. The AIR condition was room air, which was presented via the olfactometer as an additional control condition.

For both fMRI and behavioral experiments, all olfactory stimuli were presented via a MRI compatible olfactometer of our own design. The airflow control system was located outside the testing room and was based upon a Lorig-design olfactometer [63] ensuring that switching between six samples could be achieved without change in flow velocity to the nostrils (≈2.5 L/min). Five sample lines and one constant air-flow line were fed into the testing room and were connected to the nebulizer box close to the head of the participant. The stimulus delivery was achieved through nebulizing the aqueous sweat samples in commercially available nebulizers close to the head of the participant. When pressurized, the nebulizer creates a fine mist that rapidly releases semi-volatile components from the aqueous solution into the air. We inserted one-way valves between the nebulizers and the air-mixing manifold to ensure that there was no odor leakage from nebulizers that were not currently pressurized. After mixing of the constant air-flow line (0.5 L/min) with air from one of the nebulizers in the manifold, the air was delivered through a nasal cannula to the participant.

For both fMRI and behavioral studies, we employed breathing cues synchronized to the delivery of the olfactometer. The breathing cues were continuous throughout the experiment and did not indicate the presence or absence of a condition, nor did they distinguish between conditions. Since all stimuli were delivered via a nasal cannula and we did not want to alert participants that a condition was being presented, participants were instructed and trained to breathe on cue and only through their noses for the entire duration of the experiment.

For both fMRI studies, the cue was visual and presented on a projected video screen within the scanner. This consisted of an expanding and contracting ring (Figure 1a), which cued inhalation and exhalation respectively. For the duration of the entire experiments, breathing was paced to a 5 second cycle: 2.5 seconds inhalation and 2.5 seconds exhalation, synchronized to the TR of the scan (Figure 1b). In order to verify that participants were, in fact, capable of complying with the breathing cues for the duration of the experiment, for the first fMRI experiment we monitored breathing in real time throughout the scan with a MR-compatible respiration belt (Philips Medical Systems, N.A., Bothell, WA). Respiration data were acquired at 500 Hz and noise was removed from the data using a 5th order low-pass elliptic filter with a 2 Hz cutoff. Filtered data were then passed through a peak detection algorithm, in order to calculate breathing rate and depth for each run. Because the breathing cues that we used for the fMRI experiments were visual, and therefore might have distracted from the visual task, for the behavioral experiment we instead used auditory breathing cues to synchronize inhalation with the olfactometer. Continuously increasing and decreasing pitch, combined with a subtle rhythmic element that conveyed time, signaled inhalation and exhalation respectively. Respiratory compliance was monitored remotely throughout the experiment (Biopac Systems, Goleta CA).

### Validation of Trial-Specific Respiratory Parameters

To confirm compliance, we collected and analyzed respiratory data from participants tested on the first fMRI study. Each of the 16 participants had a total of 6 runs, or 96 runs total. For compliance to inhalation synchronization, 91.7% of all runs had 100% compliance to the breathing pattern, 4.2% had 95–99% compliance, 2.1% had 90–94% compliance, and 2.1% had 85–89% compliance. No run had less than 85% compliance. Performing paired t-tests between test and control conditions, there were no differences in respiratory compliance for either rate (*stress−exercise*: *t* = −1.03, *p* = 0.32, N = 16) or depth (*stress−exercise*: *t* = −0.81, *p* = 0.43, N = 16).

### General Inclusion/Exclusion Criteria for Detector Participants

The aim of the experiments was to investigate the effect of STRESS vs. EXERICISE sweat on unrelated individuals (“Detectors”). There was no overlap for the 144 Donor and 46 Detector participants, nor was there overlap, for either Donors or Detectors, between either of the fMRI experiments and the behavioral experiment. All potential Detector participants for the fMRI, odor perception, and behavioral studies, were screened for total or partial anosmia and nasal congestion using the University of Pennsylvania Smell Identification Test (Psychological Assessment Resources, Lutz FL). Due to the possible interactions between hormonal changes and olfaction [64]–[67], female participants were excluded if they were pregnant or lactating and were not tested during menstruation; additionally, the Smell Identification Test was administered prior to the scan because of hormonal variability during the menstrual cycle.

### Methods for fMRI Experiments

#### Original fMRI Experiment

Olfactory stimuli were obtained from 40 male Donors. Detector participants were 16 males and females (50% female). Detector participants were between the ages of 18 and 27 (μ = 22, s.d. = 3), and were excluded if they had a history of mental illness or substance abuse [68], neurological illness, claustrophobia, or metal in the body.

Six runs of 104 repetitions (4:20) were performed. In each run, there were six conditions presented (STRESS, EXERCISE, and AIR), each presented once during continuously synchronized closed-mouth nasal breathing and once during which the participant was instructed to sniff. Our analyses showed that the breathing and sniffing conditions generated very distinct time-series, with sniffing producing quickly decaying time-courses more typical of olfactory processing [69]–[71], and breathing producing longer time-courses more similar to the standard 15–20 s hemodynamic response function associated with non-olfactory stimuli. The two conditions could not be analyzed together, since the breathing conditions required analyses with a standard HRF while the sniffing conditions required analyses with a short (5 s) olfactory HRF [69]. Like the breathing condition, the sniffing condition also significantly activated the amygdala (left amygdala SVC: *t* = 3.45/*Z* = 2.92, *p*_uncorr_ = 0.002, *p*_corr_ = 0.08, N = 16; ROI superficial left amygdala *t* = 2.27/*Z* = 2.07, *p* = 0.02, N = 16; ROI whole left amygdala *t* = 2.10/*Z* = 1.93, *p* = 0.03, N = 16; right amygdala SVC: *t* = 4.87/*Z* = 3.71, *p*_uncorr_ = 0.000, *p*_corr_ = 0.03, N = 16; ROI superficial right amygdala *t* = 0.81/*Z* = 1.35, *p* = 0.22, N = 16; ROI whole right amygdala *T* = 1.41/*Z* = 0.79, *p* = 0.09, N = 16). In this paper we focus on the breathing conditions, since their time-course is more clearly similar to those produced by emotional, rather than olfactory, stimuli; a separate article will address the sniffing conditions. Each odor period lasted 20 seconds (4 TR), with a 20 second gap between odor presentations (REST). The orders of the six conditions were pseudo-randomized in each of the six runs. Besides the visual inhalation cues and three odor conditions (stress sweat, exercise sweat, and air), no other stimuli were presented to participants during the scans.

Participants were told that they might or might not smell odors throughout the experiment. They had only one task; this was to follow the breathing cues, which were continuous throughout the experiment.

Data were acquired using a Philips 3T Achieva whole body scanner (Philips Medical Systems, N.A., Bothell, WA) with an eight-channel SENSE™ head coil. After an initial localizer scan, a high resolution T1-weighted MPRAGE3D anatomical image (TR/TE = 8.0/4.3 ms, flip angle = 18°, FOV = 250×250×150 mm, 256×256×168 matrix) was acquired for anatomical registration. All fMRI data were collected as follows: single shot gradient echo EPI, TR/TE = 2500/22 ms, 96×96 matrix, 224×224 mm FOV, 36 interleaved transverse slices (aligned to the AC-PC line) 3.5 mm thick with no gap, 1 average, flip angle = 83°. Iterative optimization of all acquisition parameters prior to the experiment ensured that the mean intensity for signal to noise was adequate over our region of interest, the amygdala (*left amygdala*: 179.4; *right amygdala*: 175.0).

All image pre-processing for the analyses was implemented using the SPM5 program (Wellcome Department of Cognitive Neurology). For each participant's GE-EPI dataset: 1) Data were temporally shifted to correct for the order of slice acquisition, using the first slice acquired in the TR as the reference. 2) All GE-EPI images were realigned to each other. 3) The T1-weighted (structural) image was co-registered to the first EPI volume using a mutual information co-registration algorithm. 4) The co-registered high-resolution image was used to determine parameters (7×8×7 non-linear basis functions) for transformation into a Talairach standard space defined by the Montreal Neurologic Institute template brain supplied with SPM5. 5) This transformation was applied to the GE-EPI data, which were re-sliced to 2 mm×2 mm×2 mm using 7^th^ degree polynomial approximation to sinc-interpolation. 6) The spatially normalized GE-EPI data were spatially smoothed with an isotropic Gaussian kernel (full-width-at-half-maximum = 6 mm).

The fMRI data analysis comprised two levels of voxel-wise General Linear Models (GLMs). The first-level GLM is a participant-separable time series analysis that yields summary measures to be used in the second-level GLM, which affords statistical inference at the population level. In the first-level GLM, conditions were modeled with predictors comprising 20-second duration boxcars convolved with the default hemodynamic response function (HRF) of SPM5. A 0.2 Hz signal (to model the breathing frequency), motion parameters and their squares were included in the model as nuisance covariates. Linear combinations of the estimated coefficients of these predictors (i.e., contrasts) of interest were then computed per voxel per participant. These contrasts were: (1) STRESS−EXERCISE; (2) STRESS−AIR; and (3) EXERCISE−AIR. The first-level contrast images were used as the dependent variables into second-level GLMs. For each contrast, we performed a second-level statistical parametric mapping (SPM) test that was hypothesis-driven and therefore restricted to the amygdala bilaterally. The threshold for this test was obtained by using Gaussian random field small volume correction (SVC), as implemented in SPM5, with the search volumes defined by using the publicly available region of interest library, the Anatomical Toolbox [72], for the amygdala and its sub-divisions.

### Replication fMRI Experiment

Olfactory stimuli were obtained from 40 Donors (50% female). Detector participants were between the ages of 18 and 50 (μ = 26, s.d. = 3), and were excluded if they had a history of mental illness or substance abuse [68], neurological illness, claustrophobia, or metal in the body.

Based upon our results from the previous fMRI experiment, we had participants breathe continuously throughout this experiment, with no sniffing conditions; this significantly increased statistical power by effectively doubling the number of trials we could obtain from the same amount of sweat. Four runs of 144 repetitions (6:00) were performed. In each run, there were three conditions presented (STRESS, EXERCISE, and AIR). STRESS and EXERCISE were each presented four times; air was presented between each of the conditions (REST), as well as once at the end of each run as a separate condition (AIR). Since we wanted to determine whether there were gender effects, either for donor sex, detector sex, or donor*detector sex, in half of the STRESS conditions we used male donor stress sweat, and in the other half we used female donor stress sweat, with runs counter-balanced for order between participants. Each odor period lasted 20 seconds (4 TR), with a 20 second gap between odor presentations (REST), identical to the AIR condition. Besides the visual inhalation cues and three odor conditions (stress sweat, exercise sweat, and air), no other stimuli were presented to participants during the scans.

Data were acquired using a Siemens 3T Trio whole body scanner (Siemens Medical Systems, Malvern, PA) with a circularly polarized T/R head coil. After an initial localizer scan, a high resolution (T1 weighted MPRAGE3D, resolution (RL, AP, SI) of 1.33×1×1 mm (TI = 1100, TR/TE = 2100/2.74, α = 120, FOV = 170×256×256 mm, 128×256×256 pixels, total imaging time 8:59) was acquired for anatomical registration. All fMRI data were collected as follows: single shot gradient echo EPI, TR/TE = 2500/30 ms, 64×64 matrix, 224×224 mm FOV, 26 interleaved transverse slices (aligned to the AC-PC line) 3.5 mm thick with no gap, 1 average, flip angle = 83°.

The fMRI data were pre-processed and statistically analyzed as described in the original experiment.

### Methods for Odor-Perception Experiments

#### Assessment of Odor Intensity and Valence

Olfactory stimuli were obtained from the 80 Donors (25% female) who provided samples for the two fMRI experiments. Detector participants were the last 10 participants who participated in the first fMRI experiment, as well as the 16 participants who participated in the second fMRI experiment (N = 26, 50% female). Immediately following the fMRI acquisition, we performed structured assessments of odor perception; both participants and researchers were blind to vial contents, and conditions were presented randomly. Participants were provided the vials one at a time and asked to rate them individually for strength and pleasantness on Likert scales between 0 (no detectable odor/extremely pleasant) to 10 (extremely strong odor/extremely unpleasant). Scores for all three stress-sweat vials and all three exercise-sweat vials were averaged for the statistical analyses, which used non-parametric Wilcoxon Sign-Ranks tests.

#### Forced Choice Odor-Discrimination Task

Olfactory stimuli were obtained from the 64 Donors (50% female) who provided samples for the behavioral study. Detector participants were 14 individuals who participated in the behavioral experiment, as well as an additional two individuals (N = 16, 50% female; μ_age_ = 24, s.d. = 5). Each participant received 16 discrimination trials in a same-different paradigm, with 8 different-odor pairs and 8 same-odor pairs presented in random order. The odorants were fragrance paper strips dipped for three seconds in either stress sweat or exercise sweat. The participants were told that they would be presented with two substances for each trial, and were instructed to indicate whether the odorants smelled “the same or different.” The paired odorants in each trial were presented in quick succession within an interval of a few seconds. A minimum of 10 seconds was allowed between trials. Participants were allowed to sniff only once for each presentation and were then required to respond either “same” or “different”, or to guess if unsure. No feedback was given as to the correctness of the response; the odor assessment was performed with both participant and researcher blind to condition. Accuracy scores were computed for each participant, and statistically compared to chance (50%) using a one-sample t-test.

### Methods for Behavioral Experiment

Olfactory stimuli were obtained from 64 Donors (50% female). Detector participants were 14 individuals (5 female; μ_age_ = 23, SD = 5) with no history of mental illness, substance abuse [68], or neurological illness. All visual stimuli were obtained from the Pictures of Facial Affect (Paul Ekman Inc., Oakland CA). Using commercially available software (MorphMan 3.0, STOIK Imaging, Moscow Russia), we produced nine levels of morph (10%–90%) equally distributed between the Neutral (0%) and Angry (100%) poles. Pilot testing prior to the experiment (N = 8, 50% female) established reliability curves for all faces in the set; from these we selected the three faces (EM, JJ, PE; all male) that, without olfactory stimuli, most reliably produced classically-psychometric responses along morph levels. All visual stimuli were presented on a 42-inch plasma screen situated 6 feet from the participant, in an otherwise dark and silent audiometric chamber. As illustrated by Figure 5a, each trial consisted of a 500 ms of rest, 2500 ms of inhalation, a brief stimulus-on period (200 ms), 500 ms of rest, and 2500 ms of exhalation (total of 5700 ms per trial). Pilot studies with 4,16-androstadien-3-one, oestra-1,3,5(10),16-tetraen-3-ol, as well as common odorants, were conducted prior to the experiment in order to optimize experimental parameters; these were set at 14 trials per 9 morph levels, with morph levels and faces chosen randomly for each trial. These 126 trials (14×9 morph levels) were presented under two conditions: while breathing stress sweat (STRESS), and while breathing exercise sweat (EXERCISE). The total experiment ran for 28 minutes. To maximize participant compliance and focus throughout the experiment, testing was divided up into four seven-minute runs: two for the STRESS condition, and two for the EXERCISE condition, with two minutes between runs. STRESS and EXERCISE conditions alternated for each participant, with condition order counter-balanced between participants.

Participants were instructed to indicate, following presentation of the facial stimuli, whether the face was “more neutral or more threatening.” Participants used a two-button computer mouse to make their choice, and were asked to do so as quickly as possible without making errors. Limits were set so that responses were not accepted after 2500 ms post-presentation; responses provided in fewer than 200 ms were excluded.

Each individual participated in 5 minutes of training prior to the experiment. Participants first practiced following the auditory breathing cues alone, using only room air. Participants then practiced only using the mouse to identify the faces as neutral or threatening. For the practice sessions, we used 0% angry and 100% angry poles for the three faces presented in the experiment; this served to perceptually “fix” the endpoints for the psychometric curves for all participants. Finally, after mastering both of these components, participants combined them to practice the behavioral task with the breathing cues, again using only 0% and 100% angry faces and room air.

This experiment was optimized for psychometric curve-fitting, a method often employed to exploit the instability linked to ambiguous stimuli in order to test subtle shifts in perception induced by an external manipulation [73]. For each participant, data for each condition (STRESS, EXERCISE) were fitted with a sigmoid function
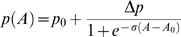
(3)where *p_0_* and *p_0_+Δp* define upper and lower asymptotes, *A_0_* was the inflection point, and *σ* defined slope. The fitting was done using curve-fitting toolbox packaged with Matlab 7.4.0. (Mathworks, Natick MA). This function provided a natural fit to the data with a mean R^2^ value of 0.98±0.02, as compared to a simple linear fit with mean R^2^ value of 0.89±0.06. Statistical tests (repeated-measures ANOVA) were then performed on individual inflection points and slopes for each participant to assess changes in perceptual threshold and discrimination, respectively.
